# Synergistic potentiation of the anti-metastatic effect of anti EGFR mAb by its combination with immunotherapies targeting the ganglioside NGcGM3

**DOI:** 10.18632/oncotarget.25290

**Published:** 2018-05-08

**Authors:** Addys González Palomo, Armando López Medinilla, Valeria Segatori, María del Carmen Barroso, Rances Blanco, Mariano R. Gabri, Adriana Carr Pérez, Kalet León Monzón

**Affiliations:** ^1^ Center of Molecular Immunology (CIM), Atabey, Playa, Havana, Cuba; ^2^ Laboratory of Molecular Oncology, Quilmes National University, Buenos Aires, Argentina

**Keywords:** combined therapy, metastasis-models, survival, signaling pathway, NGcGM3

## Abstract

Several Anti-EGFR mAbs are register for the treatment of human cancer. However, their impact on patients overall survival has been limited by tumor resistance. *N*-Glycolyl variant of GM3 ganglioside (NGcGM3) is specifically expressed in some human tumors, and it has been associated with a poor prognosis. Several reports have documented that GM3 physically associates to EGFR inhibiting its ligand depend phosphorylation, but it also facilitates an alternative/compensatory signaling cascade mediated by Uroquinase Plasminogen Activator Receptor (uPAR) and integrin α5β1 interaction. However, the difference between NGc and *N*-Acetylated (NAc) variants of GM3 regarding such interactions is unknown. We hypothesized that enrichment of NGcGM3 expression in tumors relates to advantages of this ganglioside, on ensuring both EGFR and uPAR pathways optimal function. We explored the impact of combining an anti-EGFR (7A7 mAb) with anti-NGcGM3 therapies: NGcGM3/VSSP vaccine or 14F7 mAb. Both combinations synergistically increase overall survival in two models of lung metastasis: 3LL-D122 and 4T1; but combination with NGcGM3/VSSP vaccine is significantly more effective. In 3LL-D122-metastasis, of mice treated with the best combination, both EGFR and uPAR/α5β1 integrin pathways are turn off (I.e expression of uPAR/α5β1; and phosphorylation of EGFR, Stat3, Src and FAK are reduced); and tumor angiogenesis is decreased. Interestingly, combination treatment increases tumor infiltrating CD4^+^T, CD8^+^T and NK^+^-cells. Furthermore, a positive clinical outcome is reported for a cancer patient treated with an anti-EGFR mAb and anti-NGcGM3 therapy. Overall, our results support the combination of anti EGFR antibodies with therapies targeting NGcGM3 to increase their efficacy in future clinical trials.

## INTRODUCTION

Around 90% of all deaths related to cancer are the result of metastasis rather than primary tumors. Metastasis is a multistep complex process and no single therapeutic approach targeting one single pathway has so far provided good results in term of overall survival [1, 2]. EGFR plays an important role in cancer progression. Activation of EGFR results in the phosphorylation of several downstream proteins, which promote cell proliferation, invasion and metastasis [3]. Several anti-EGFR therapeutic approaches have demonstrated antitumor effect [4–6], however; clinic benefits in terms of overall survival has been limited. Diverse strategies based on anti-EGFR antibodies, like Cetuximab [7] and Nimotuzumab [5] induce tumor shrinkage or stabilization, mainly in combination with chemotherapy or radiotherapy, but resistant tumor variants rapidly emerge. This fact seriously limits anti-EGFR impact on patient overall survival. Resistant tumor variants frequently show activation of alternative signaling pathways that stimulate tumor proliferation [7–10].

Gangliosides are frequently over-expressed in tumors in comparison with the corresponding normal tissues [11, 12]. They have an important role in tumor progression and metastization [11, 13]; they modulate signaling through different receptors in normal and tumor cell membranes [10, 14]. Gangliosides are one of the immunosuppressive molecules released by tumors to their microenvironment [13, 15]. NGcGM3 has been recognized as an effective target for cancer therapy [15–17]. This ganglioside is over-expressed in several tumors [18–20], and once shed to the microenvironment impairs CD4^+^ T cells and dendritic cells function [15]. Recently, NGcGM3 expression has been associated with a poor prognostic in colon [19] and non-small cell lung cancer (NSCLC) [21]. Multiple clinical trials have been performed with the NGcGM3-containing vaccine, developed at the Center of Molecular Immunology (CIM) for melanoma [22] and breast cancer [16, 23] showing evidences of clinical effect.

Several reports in the literature have documented a functional relationship between the ganglioside GM3 and EGFR in the tumor cell membrane. GM3 physically binds to EGFR inhibiting its ligand-dependent phosphorylation [9, 24], but it triggers an alternative/compensatory signaling cascade mediated by the interaction of Uroquinase Plasminogen Activator Receptor (uPAR) and the α5β1 integrin [25–27]. Most data available, in this respect, corresponds to experiments performed with the most abundant NAc variant of GM3, and little information exists on the modulation by NGcGM3 of EGFR and uPAR/α5β1 integrin pathways. A recent *in vitro* study demonstrated that EGFR phosphorylation in A431 cell lines is less inhibited in the presence of NGcGM3 than in the presence of NAcGM3 [24]. Moreover, co-expression of NGcGM3 and EGFR is a relatively frequent phenomena in cancer patients [28] and what it is more relevant the over-expression of both EGFR and NGcGM3 variant correlates with a worst prognostic in NSCLC patients [21] than the overexpression of either of these molecules separately.

We hypothesized that the accumulation of NGcGM3 in the cell membrane, ensures optimal EGFR and uPAR/α5β1 integrin pathways signaling, promoting tumor growth and metastasis dissemination. NGcGM3 over-expression could make tumors less dependent of EGFR phosphorylation and therefore less sensible to anti-EGFR based therapies. For instance, NGcGM3 accumulation could increase signaling through uPAR/α5β1 integrin and/or other alternative pathways. To address this hypothesis, we explore the impact of combining an anti-EGFR mAb with either active (NGcGM3/VSSP vaccine) or passive (14F7 mAb) therapies targeting NGcGM3. In mice, both combinations synergistically increase overall survival in two models of lung metastasis; but the combination with NGcGM3/VSSP vaccine was significantly more effective. In the metastasis, of mice treated with the combination, both EGFR and uPAR/α5β1 integrin pathways are turn off; and tumor angiogenesis, a process influenced by these pathways, is reduced. Interestingly combination treatment increases tumor infiltrating CD4^+^T, CD8^+^T and NK^+^ cells and its therapeutic effect is abrogated by the *in vivo* depletion of any of these cell populations. Furthermore, a positive clinical outcome is reported for a cancer patient treated with the combination of an anti-EGFR mAb and anti-NGcGM3 therapy. Overall, our results support the combination of anti-EGFR antibodies with therapies targeting NGcGM3, as an alternative, to increase their efficacy in future clinical trials.

## RESULTS

### Synergic effect of immunotherapies targeting EGFR and NGcGM3 on spontaneous lung metastasis models

EGFR and NGcGM3 ganglioside are molecules co-expressed on spontaneous lung metastases induced by 3LL-D122 or 4T1-clones [28]. Interestingly in the 3LL-model, NGcGM3 expression increases progressively from the primary tumor to the lung metastasis [29], being almost inexistent on the tumor cells when cultured *in vitro*. Additionally, both targets are co-localized in lung metastases generated by these two tumor models (Supplementary Figure 1).

To study the combination we used the 7A7 mAb (anti-EGFR therapy) [30]. This mAb has some effect on the formation of lung metastasis, but it does not improve mice overall survival. This behavior is similar to what happens in the clinic with some anti-EGFR mAbs. They slow down tumor progression, but they do not increase patients overall survival. The resistance of 3LL-D122-cells to the treatment with 7A7 mAb has been extensively characterized [31]. Resistant 3LL-D122-tumor variants exhibit a quite diverse range of escape mechanisms [31]. As a first anti-NGcGM3 therapy, we used the NGcGM3/VSSP vaccine [29] which is quite immunogenic in mice [29] and humans [17, 22]. This vaccine has shown anti-metastatic effect in the 3LL-D122 spontaneous metastasis model [29], but it does not increase the overall survival of mice.

**Figure 1:**
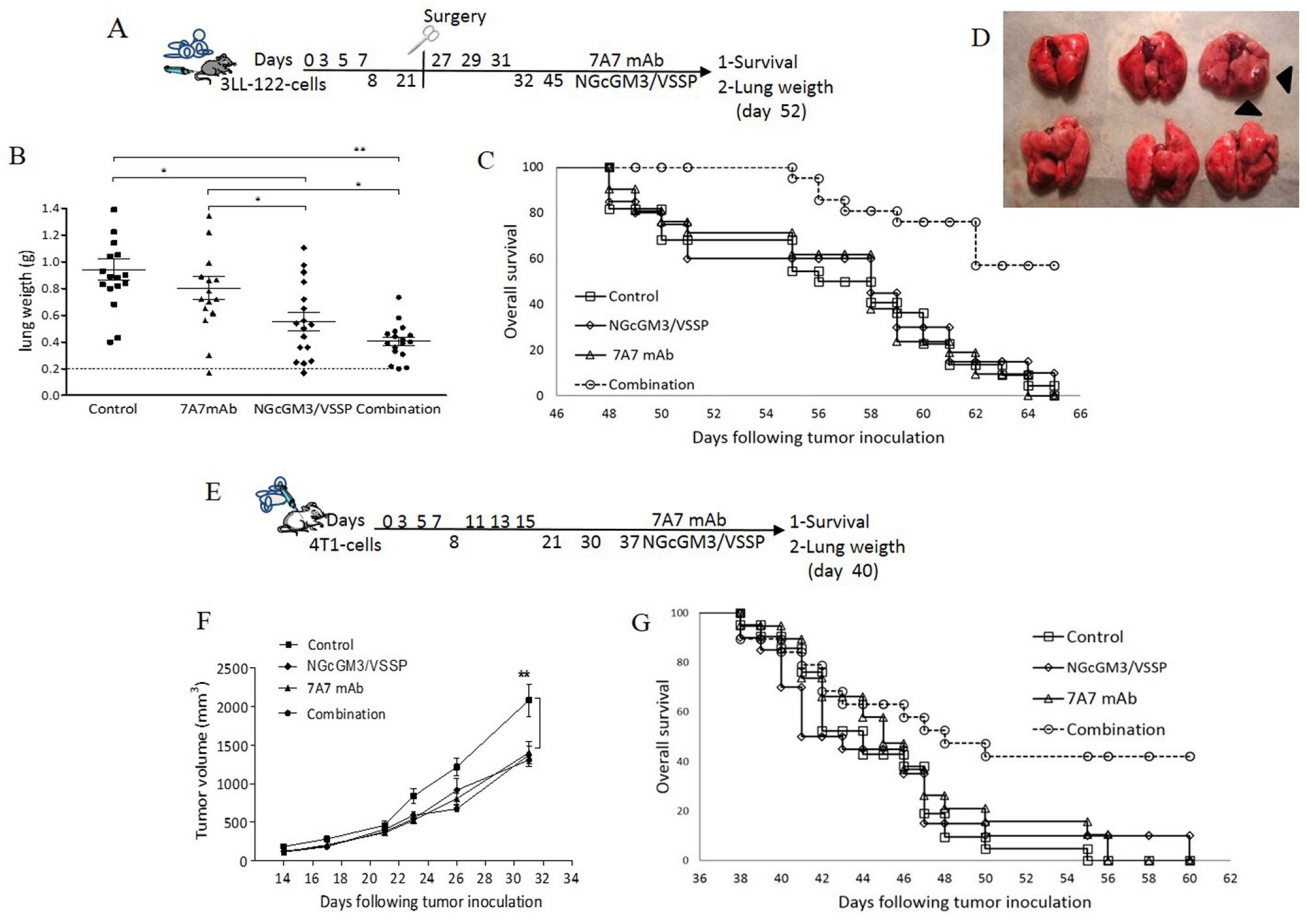
Combinatorial-targeted therapy to EGFR (7A7 mAb) and anti-NGcGM3 (NGcGM3/VSSP vaccine) increase the survival of mice bearing spontaneous metastases 3LL-D122-model **(A-D)**. 4T1-model **(E-G)**. The treatment with PBS, 7A7, NGcGM3/VSSP vaccine or Combined therapy is indicated in schematic representation of 3LL (A) or 4T1-models (E). Lung weight in 3LL-model (B). Normal lung weight value is indicated (dashes lines). Each group represents mean±SD of lung weight/animals. Primary tumor volume in 4T1-model (F). Each point represents mean±SD per animals/group. To analyze the percentage of survival, animals (n=10/group) were monitored every day. Kaplan-Meier curves of overall are showed for 3LL (C, p<0.0013) and 4T1 (G, p<0.04) models, (Log-rank test). Lungs of surviving mice show the anti-metastatic effect of the combination therapy (D). One representative experiment out of three performed experiments is shown in each case.

Figure 1A–1D shows the effect of the combination of NGcGM3/VSSP vaccine and 7A7 mAb in the model of spontaneous lung metastasis induced by 3LL-D122-cells. Figure 1A indicates the experimental scheme. Figure 1B illustrates the lung weight in an earlier cut off the experiment by day 52. Overall, the combination does not affect the growth of the primary tumor (Supplementary Figure 1I); reduces the number of lung metastases to a similar extend than the NGcGM3 vaccine alone (Figure 1B); but it synergistically increased the mice overall survival (Figure 1C). Interestingly, while the individual therapies do not increase overall survival, in the combination group 60% of the mice remains alive after all mice in control group are dead. Moreover, careful analysis of the lungs of survivor mice by day 71, shows a strong anti-metastatic effect of the combination, only 2 out of 6 mice have any detectable metastases (Figure 1D).

Figure 1E–1G shows the effect of the combination of NGcGM3/VSSP vaccine and 7A7 mAb in the spontaneous lung metastasis model induced by 4T1-cells. Figure 1E indicates the experimental scheme. Figure 1F presents the growth kinetics of the primary tumor. Supplementary Figure 1J exhibits the lung weight in an earlier cut off the experiment by day 40. Overall, the combination reduce the growth of the primary tumor, similarly to the NGcGM3 vaccine and 7A7 individual treatments (Figure 1F); but it synergistically increased the mice overall survival (Figure 1G). Interestingly, as in the 3LL-D122 model, while the individual therapies do not increase survival, in the combination group 40% of the mice remained alive (but not tumor free) after all mice in the control group are died. Such increase in mice overall survival most likely reflects a delayed anti-metastatic effect of the combination (manifested only after day 40).

Additionally, we substituted the ganglioside vaccine for a passive therapy with the 14F7 mAb, which is specific for NGcGM3 ganglioside [32]. Previously, we reported that the combination therapy using the mAbs 7A7 and 14F7 increases the overall survival on the models of experimental lung metastasis of 3LL-D122 [28]. Here we further evaluated this combination of passive therapies, but in the spontaneous lung metastasis model (3LL-D122-cells) [28]. We found that 30% of the mice consistently remain alive only the combination group. The overall survival curves are shown in Supplementary Figure 1K. It should be noted that the impact of this combination is clearly smaller than the one reported on Figure 1C.

### Combination turns off signaling by the EGFR and uPAR/α5β1 integrin on lung metastasis

Next, we compare the level of activation of both EGFR and the uPAR/α5β1 integrin pathways on the 3LL-D122 metastases extracted at day 52, from mice treated according to the schemes shown in Figure 1A. We use FACS and immunofluorescence (IF) assays to detect expression and/or phosphorylation state of several molecules in these pathways.

**Figure 2:**
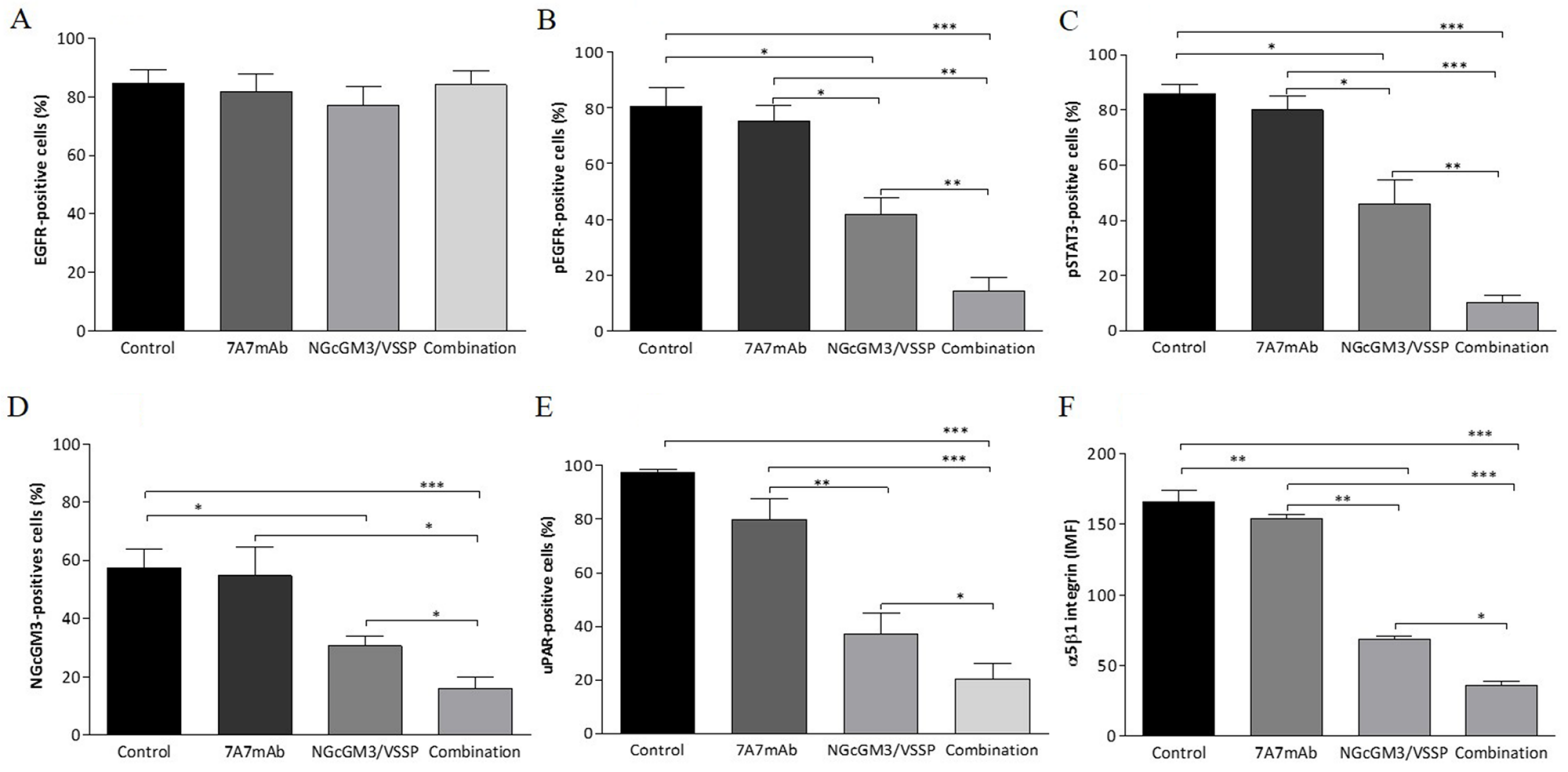
Combinatorial-targeted therapy to EGFR (7A7 mAb) and anti-NGcGM3 (NGcGM3/VSSP vaccine) simultaneously turns off signaling by the EGFR and uPAR/α5β1 integrin on remaining 3LL-metastases 3LL-model and the treatments as described in Figure 1A. Tumor cells from 3LL-metastases post-treatments were obtained to determine the expression/activation of EGFR **(A)**, p-EGFR (fosfo-EGFR, **B**), p-Stat3 (fosfo-Stat3, **C**), NGcGM3 ganglioside **(D)**, uPAR **(E)** and α5β1 integrin **(F)** by flow cytometry. The bar diagrams shows the percentage of positive cells in each case. Data are represented as mean±SD. (n=6). Statistical analysis was performed using Two-way ANOVA, combined with the Kruskall-Wallis test, for multiples comparison was employed. Statistical differences are indicated: ^***^p<0.001, ^**^p<0.01, ^*^p<0.05. One representative experiment out of three performed experiments is shown in each case.

EGFR expression is similar for all treated groups (Figure 2A). However, NGcGM3/VSSP vaccine surprisingly reduces the phosphorylation of EGFR (Figure 2B) and Stat3 (Figure 2C) as compared with 7A7 mAb and the control (PBS). Combined therapy significantly reduces EGFR and Stat3 phosphorylation with respect to all monotherapies (Figure 2B and 2C, respectively). Furthermore, NGcGM3/VSSP vaccine, but not the mAb 7A7 decreased the expression of NGcGM3 (Figure 2D), uPAR (Figure 2E) and α5β1 integrin (Figure 2F) in contrast with the control group. However, the combination induced an even greater reduction in the expression of these molecules (Figure 2D–2F). A representative dot plot from one mice/group is shown in Supplementary Figure 2. The same results were obtained by IF assay (Supplementary Figures 3 and 4).

**Figure 3:**
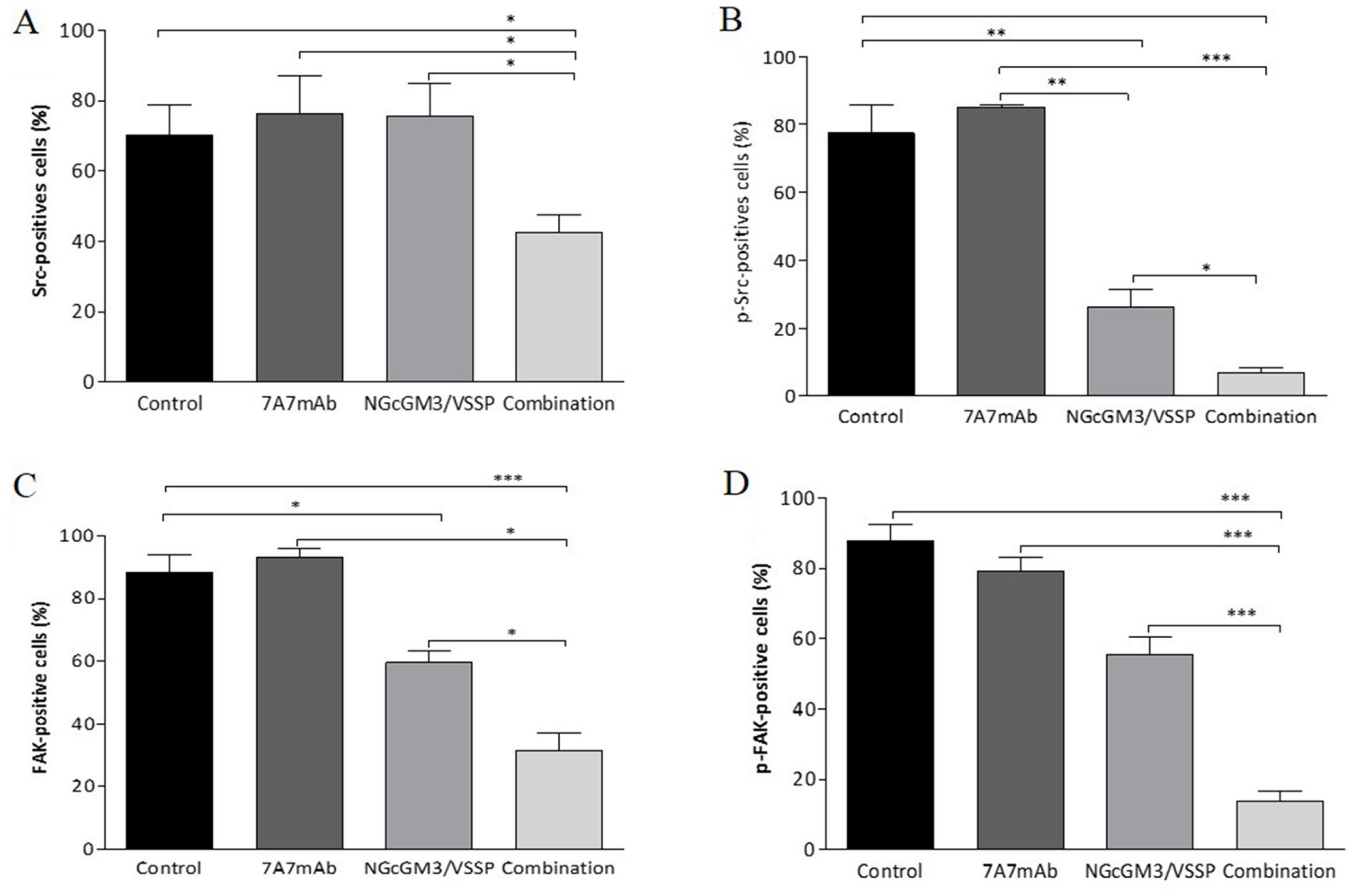
Combinatorial-targeted therapy to EGFR (7A7 mAb) and anti-NGcGM3 (NGcGM3/VSSP vaccine) simultaneously inhibits Src and FAK signaling pathways on remaining 3LL-metastases 3LL-model and the treatments as described in Figure 1A. Tumor cells recovered from 3LL-metastases post-treatments were obtained to determine the expression/activation of Src **(A)**, p-Src **(B)**, FAK **(C)** and p-FAK (fosfo-FAK, **D**) by flow cytometry. Data are represented as mean±SD (n=6). Statistical analysis was performed using Two-way ANOVA, combined with the Kruskall-Wallis test, for multiples comparison was employed. Statistical differences are indicated: ^***^p<0.001, ^*^p<0.05. One representative experiment out of three performed experiments is shown in each case.

uPAR/α5β1 integrin complex promotes the overexpression/activation of two soluble tyrosine kinase: Src and Focal Adhesion kinase (FAK) which are related to the metastatic process [33]. We assessed the changes in the intracellular Src and FAK status in 3LL-metastases removed from treated mice. The expression/phosphorylation of these two molecules was not modulated by 7A7 mAb (Supplementary Figure 2). NGcGM3 vaccine reduces only the phosphorylation of Src (Figure 3B) and the level of expression of FAK (Figure 3C). However, the combination significantly reduced expression/phosphorylation of Src and FAK, in comparison with the control group and the monotherapies (Figure 3A–3D). The representative dot plot per mice/group and the results by IF assay is presented in Supplementary Figures 2 and 5, respectively.

Overall the data in Figures 2 and 3 shows that 3LL-metastases after combination treatment have the largest reduction in expression/phosphorylation of molecules in both EGFR (pEGFR and pStat3) and the uPAR/α5β1 integrin (uPAR, Integrin, Src and FAK) pathways.

**Figure 4:**
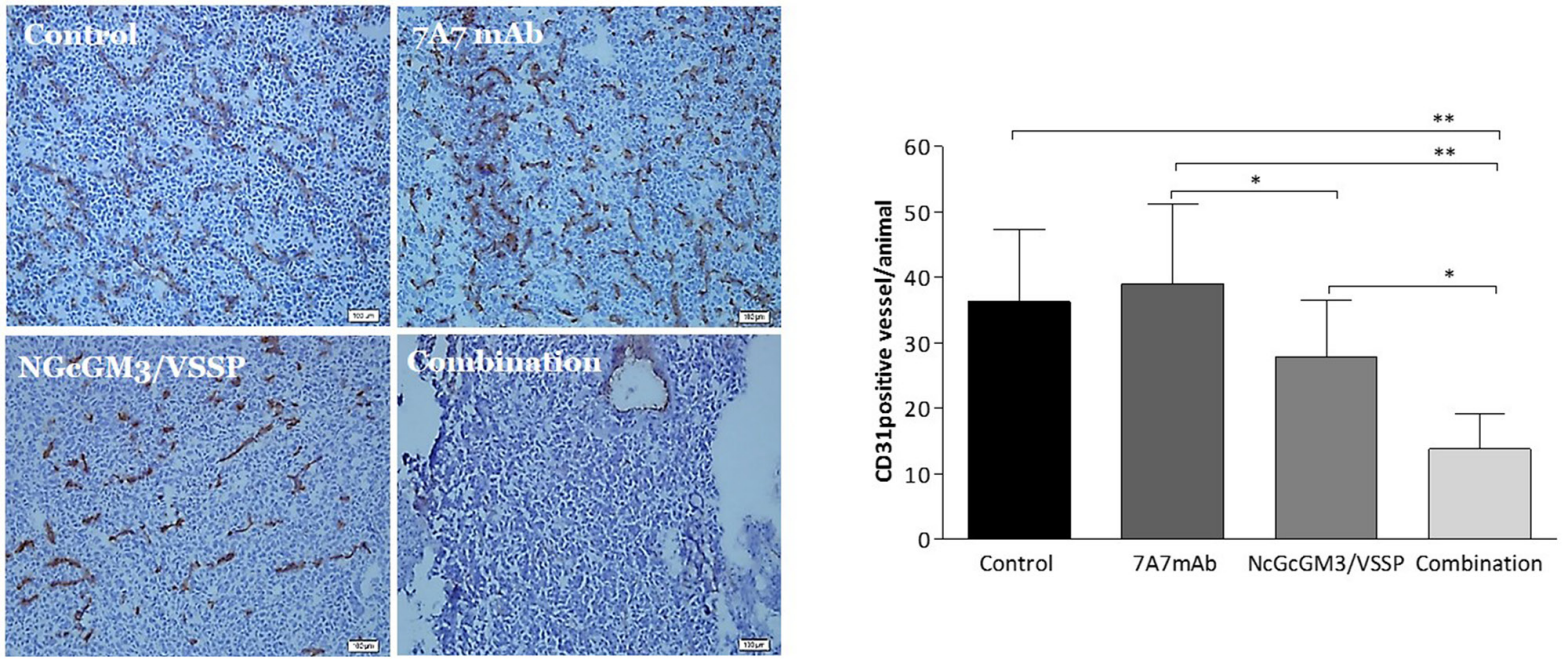
*In vivo*, intratumoral angiogenesis effects of Combinatorial-targeted therapy to EGFR (7A7 mAb) and anti-NGcGM3 (NGcGM3/VSSP vaccine) 3LL-model and the treatments as described in Figure 1A. Tumor sections from lungs were fixed with ice-cold acetone to determine the number of CD31^+^ microvessels (representative image) and quantitative results of immunohistochemical staining of angiogenic activity. Data represent the mean±SD in 10 independent 3LL-metastases fields from five mice/group. Statistical analysis was performed using Two-way ANOVA, combined with the Kruskall-Wallis test, for multiples comparison was employed. Statistical differences are indicated: ^**^p<0.01, ^*^p<0.05. One representative experiment out of three performed experiments is shown. White bars=100μm.

Finally, we assessed the functional impact of the combination therapy in 3LL-D122-metastases. Several studies have reported a relationship between EGFR signaling and uPAR/integrin signaling with the mechanisms of proliferation, survival and tumor angiogenesis [34]. We evaluated these processes by immunohistochemistry on lung metastases section of treated mice at day 52. Interestingly, after combination therapy, the number of blood vessels measured by CD31 mAb staining was significantly reduced (Figure 4). However, proliferation and apoptosis were not modified (Supplementary Figure 6A and 6B). Similarly, the combination of passive therapies reduced the number of blood vessels but not the proliferation and apoptosis in experimental 3LL-D122-model (Supplementary Figure 7).

### The synergistic effect of the combination is partially mediated by cellular immune effectors

**Figure 5:**
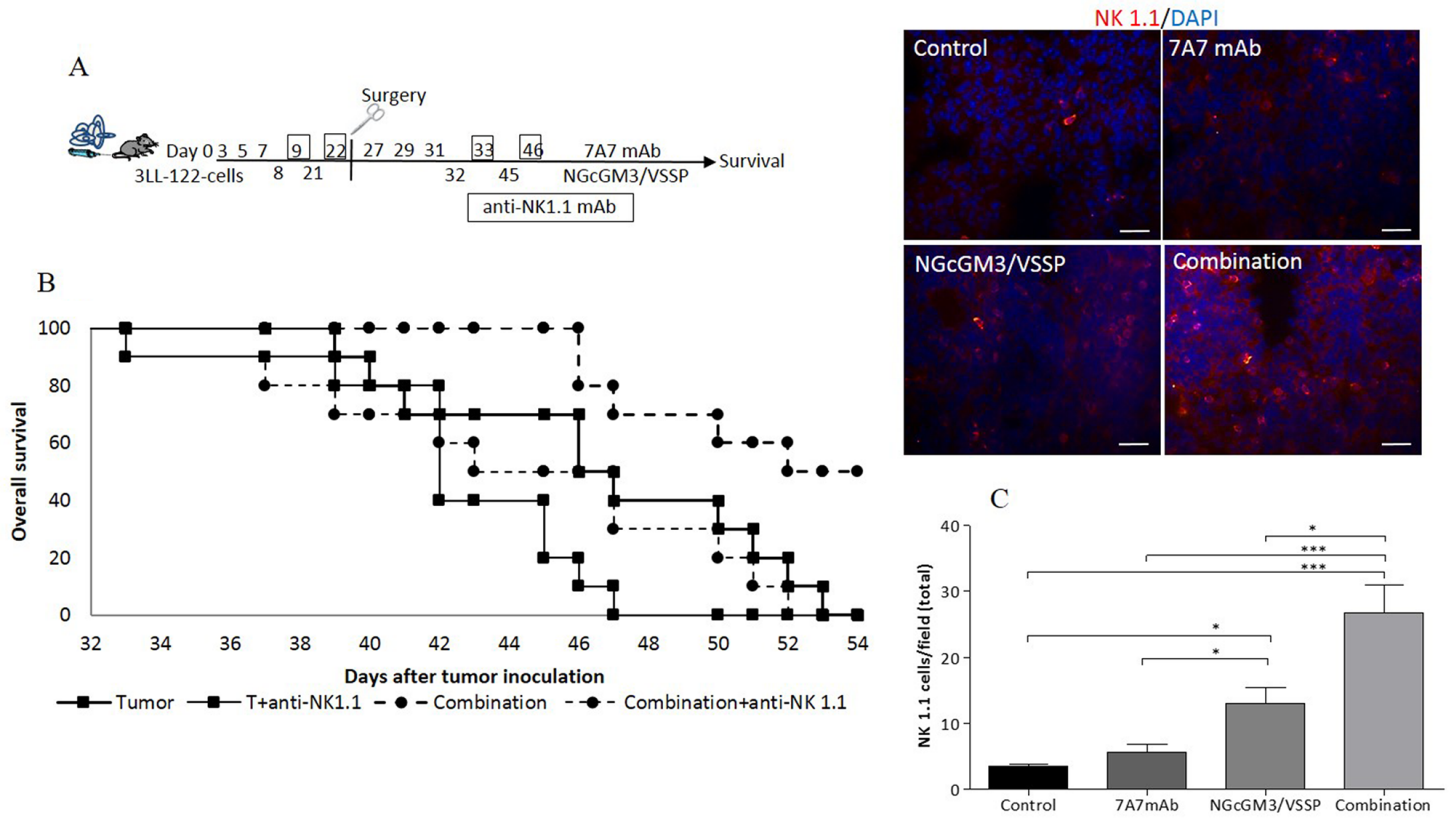
NK 1.1^+^cells are involved in increasing survival by the 7A7 mAb in combination with NGcGM3/VSSP vaccine on 3LL tumor-bearing mice 3LL-model. Administration of treatment with PBS, 7A7 mAb, NGcGM3/VSSP vaccine, combined therapy or NK 1.1 depletion is indicated in the schematic representation **(A)**. To analyze the percentage of survival, animals (n=10/group) were monitored every day. Kaplan-Meier survival curves are showed (**B**, p<0.0004). Representative photomicrographs of 3LL-metastases showing the NK 1.1^+^cells infiltration by the immunofluorescence staining. Quantitative evaluation of NK 1.1^+^cells **(C)**. Statistical analysis was performed using Two-way ANOVA, combined with the Kruskall-Wallis test, for multiples comparison was employed. Statistical differences are indicated: ^***^p<0.001, ^*^p<0.05. One representative experiment out of three performed experiments is shown in each case.

The anti-tumor activity of 7A7 mAb [35] and NGcGM3/VSSP vaccine [29, 36] depends of the induction of the cellular immune effectors, in the 3LL-D122-models. Then, we explore the contribution of CD4^+^T, CD8^+^T and NK1.1^+^cells on the synergistic effect of the combination therapy in the 3LL-D122 model. To this aim, we first individually depleted these cells in treated mice by injecting anti-CD4, anti-CD8 or anti-NK1.1 mAbs, 24h after each vaccine administration. Figure 5A indicates the experimental scheme for the case of NK1.1 cell depletion. Survival benefit of the combination therapy was completely abrogated in mice depleted of NK1.1^+^ cells (Figure 5B) or CD4^+^T and CD8^+^T cells (Supplementary Figure 8B and 8D).

As a second strategy to access the role of CD4^+^T, CD8^+^T and NK1.1^+^cells in the combination effect, their infiltrate on 3LL-metastases was evaluated. Following the same schedule of treatment presented in Figure 1A, the number of infiltrate immune cell in 3LL-metastases (at day 52) was quantified by immunohistochemistry. All treatments increase the presence of CD4^+^T and CD8^+^T cells in the tumor (Supplementary Figure 8C and 8E) as compared with the control, but no difference among them was observed. The infiltrate of NK 1.1^+^cells in the vaccine group was greater than in 7A7 mAb and the control groups (Figure 5C). But, interestingly, NK1.1^+^ infiltrate was most significantly increased in the combination group (Figure 5C). Overall, this data suggest that the cellular immune response induced indistinctly by the NGcGM3 vaccine and the 7A7 mAb mediate the synergistic effect of their combination.

### Results from a patient treated with the combination of an anti-EGFR mAb and anti-NGcGM3 therapy

Anti-EGFR antibody (Nimotuzumab) was registered in Cuba, for the treatment of several epithelial tumors mostly in combination with chemo or/and radiotherapy [37]. NGcGM3/VSSP vaccine is currently in clinical trials in melanoma and breast cancer patients [17]. These treatments both have reported low toxicity.

**Figure 6:**
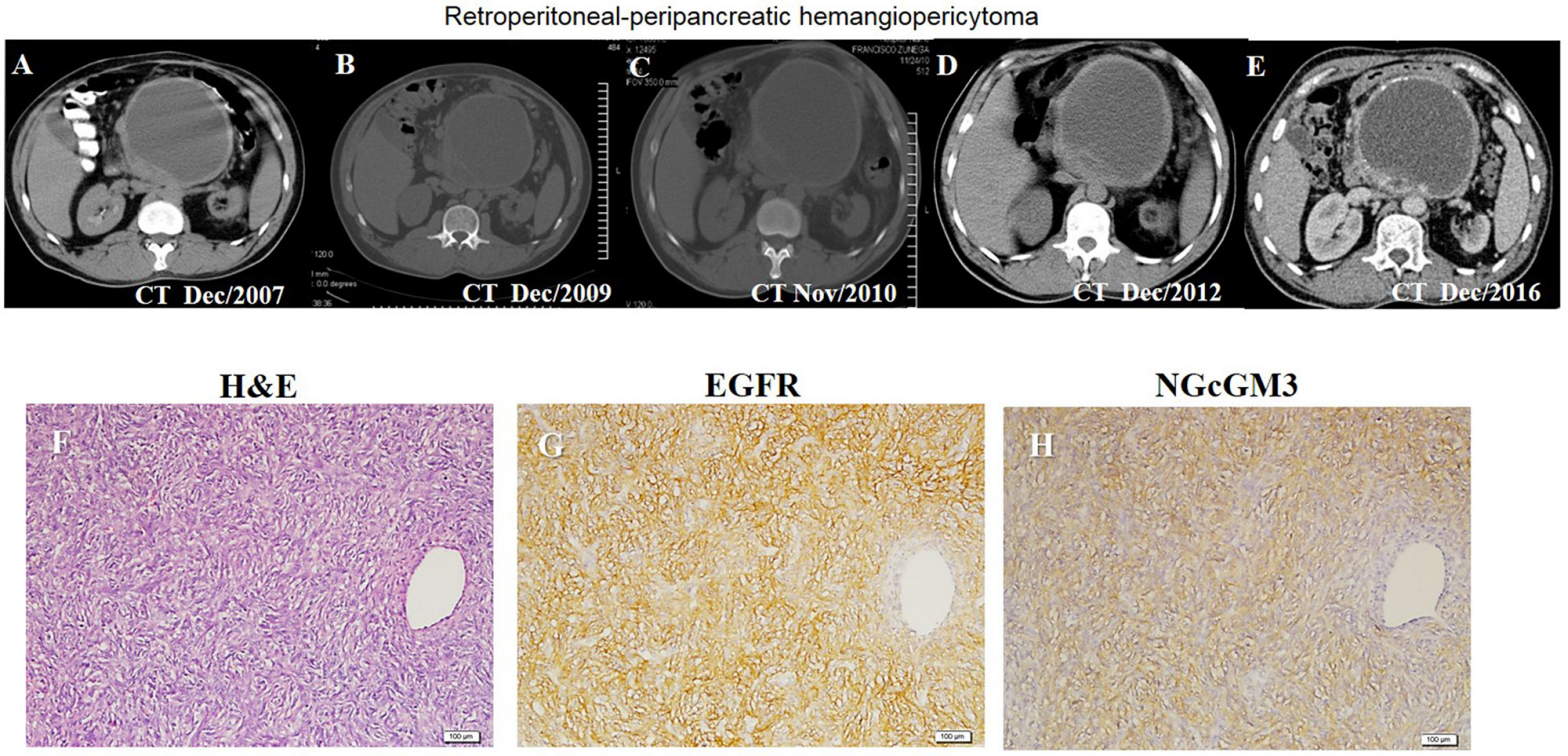
Clinical response to anti-EGFR and anti-NGcGM3 therapy in a patient with retroperitoneal-peripancreatic hemangiopericytoma Clinical response to Nimotuzumab and NGcGM3/VSSP vaccine by the sequential computed tomography during 122 months **(A-E)**. Hematoxylin and eosin (H&E)-stained tumor tissue sections **(F)**. Images of EGFR **(G)** and NGcGM3 ganglioside **(H)** expression (brown color) by immunohistochemistry assay. EGFR was identified with Ior/egf/r3 mAb and it was mainly located in the cell membrane of malignant cells. NGcGM3 ganglioside expression was identified with 14F7 mAb and it was located in cell membrane and cytoplasmatic cell. Counterstaining with Mayer’s Hematoxylin (blue color). White bar = 20 μm.

Clinical results from a patient who received the combination of Nimo with NGcGM3/VSSP vaccine under a compassionate base are reported here. Figure 6A-6E shows a sequential computed tomography of the abdomen of a patient with hemangiopericytoma without response to chemo and radiotherapy. The patient received combination therapy 18 months after the initial diagnosis. An image taken at the start time of therapies is observed in Figure 6A; Figure 6B–6E illustrate the results of treatment during 15 years, long lasting disease stabilization without changes on tumor size. Furthermore, after 122 months treatment the tumor of the patient continues to have the same size (Figure 6E). The patient maintains an excellent quality of life. High expression of EGFR and NGcGM3 was detected by immunohistochemistry assay on tumor biopsy before immune treatment (Figure 6F–6H). It is important to notice that no sign of toxicity were detected associated to the combination therapy.

## DISCUSSION

In this work, we explored the impact of combining an anti-EGFR (7A7 mAb) with either active (NGcGM3/VSSP vaccine) or passive (14F7 mAb) therapies targeting NGcGM3. In mice, both combinations synergistically increase overall survival in two tumor models of lung metastasis (Lewis lung carcinoma 3LL-D122 and mammary carcinoma 4T1); but combination with NGcGM3/VSSP vaccine was significantly more effective. Interestingly in the 3LL-model the combination with NGcGM3/VSSP vaccine virtually cured 60% of the treated mice. Specifically, only 2 out of 6 sacrificed mice have some small, but detectable metastases in the lungs. In the case of 4T1-model 40% of the mice remained alive (but not tumor free), in the combination group. The smaller effect of the combination therapy, in the 4T1-model, could be related to the lower and more variable expression of NGcGM3 on its lung metastases. However, it might also relates to the aggressiveness of this tumor model, where primary tumor always remain and metastases are frequently implanted in liver and brain, beside the lungs.

Our main hypothesis is based on the reported physical relationship between GM3 and EGFR [12, 38, 39]. Experimental data revealed that GM3 interact with EGFR inhibiting its phosphorylation, but it also interacts with the uPAR, facilitating its association with the integrin α5β1 [25–27, 40]. As a result of this interaction an alternative signaling cascade is triggered, which somehow compensate the reduction of signaling via EGFR [9]. It is important, however, to take into account the potential difference between NGcGM3 and NAcGM3 gangliosides in terms of their interaction with the EGFR and the uPAR/integrin pathways. These interactions are not well characterized for the NGcGM3. Available reports likely use the (more common) NAcGM3 ganglioside. The only report in the literature, which uses the NGcGM3, showed a lower inhibition of the EGFR phosphorylation by this ganglioside variant [24]. Furthermore, there is no information about the potential difference of NGcGM3 and NAcGM3 regarding their interaction with the uPAR and the integrin α5β1.

Here, we provide indirect evidence that the inhibition of these signaling cascades could have biological/therapeutic relevance. We found that the combination of anti-EGFR and anti-NGcGM3 therapies in the few remaining 3LL-metastases significantly turned off the signaling by means of these cascades. In these metastases, the expression of NGcGM3, uPAR and α5β1 integrin, but not EGFR, were markedly reduced, as compare with the expression in the metastases recovered from monotherapies treated mice. Moreover, the phosphorylation of EGFR, Src, FAK and Stat3 were also markedly reduced in the combination group. These results suggest that those tumor cells, which resist the combination treatment, were selected to survive in the absence of EGFR and uPAR/α5β1 mediated signals. Therefore, they shall rely on other signaling pathways to survive and proliferate *in vivo*. A more extensive studies on the direct effect of the combination therapy over the EGFR and uPAR/α5β1 mediated signals shall be carried on *in vitro* cultures. However, we are unable to do such studies at the time being, because 4T1 and 3LL-tumors loose the expression of NGcGM3 very fast after any attempt of *ex vivo* culturing. Many other epithelial murine and human tumor cells line have this behavior too. In our cell banks, only some tumors from the myeloid linage express NGcGM3. Therefore, we are unable to study the effect of combination therapy on these models too. We have also tried to exogenously incorporate synthetic NGcGM3 on 4T1 and 3LL-cells in culture. But, such incorporation happens to be quite transient and there is data in the literature [41] showing that the ganglioside is not properly integrated in the cells membrane. It does not mediate the same interactions than the endogenously synthetized NGcGM3. Future attempts might rely on transforming 3LL-D122 and 4T1-cells to express the Cmah enzyme enforcing the expression of NGcGM3, *in vitro* [42].

Interestingly, the combination of the passive therapies (7A7 and 14F7 mAbs) only partially reproduces the synergistic effect of the combination of 7A7 mAb with NGcGM3/VSSP vaccine in both tumor models. It might be expected since the binding of mAbs to their targets interferes with the interaction of these molecules in the cell membrane somehow affecting signaling through the EGFR and the uPAR/integrin cascade. The lower effect of this combination might results from a shorter persistence of the injected antibodies in circulation. The mice treated with the NGcGM3/VSSP vaccine, a strong anti-NGcGM3 antibody response is typically induced. These antibodies are mostly of the IgG isotype and could last for several months in circulation [43]. On contrast, the anti-NGcGM3 mAb passively injected have a characteristic half live of around 3 days [42].

The induction of a cellular immune response of 7A7 mAb and NGcGM3/VSSP vaccine has described, for their anti-metastatic effect in the 3LLD122-model. Not surprisingly then, the synergistic effect of their combination was fully abrogated by the *in vivo* depletion of several immune cell populations (CD4, CD8 and NK cells). The therapy with 7A7 mAb induce a CD8^+^T cells response against EGFR specific peptides [44]. This immune response depends on the immunogenic cell death induced by the mAb mediated inhibition of EGFR signaling, which likely stimulate tumor-antigen presentation by dendritic cells *in vivo*. The functional impact of such CD8^+^T cell response, in the selection of tumor resistant variants of 3LLD12-cells, has been extensively studied [31, 35]. It has been shown that the tumor frequently escape by down modulating the MHC-I molecules or other molecules of the antigen presentation machinery (APM). Some resistant tumor variants have soft (interferon reversible) MHC-I down-modulation, but other have hard (interferon irreversible) down-modulation [45]. Interestingly, MHC-I down-modulation has been classically associated with increased sensibility to the attack of NK cells. Thus, since the NGcGM3/VSSP vaccine has been shown to strongly stimulate the NK/NKT cell in the 3LL-model, it is possible that the natural complementation of the cellular response induced by these treatments is important in the synergism observed on their combination. In addition, a significant increase on the NK1.1^+^T cells infiltrates on the tumors of mice treated with the combination was observed. Although, the infiltrates of CD4^+^T and CD8^+^T cells also increases in the combination treatment, however there are no significant differences with the monotherapies. Understanding how the combination treatment specially potentate the NK1.1^+^cells response and whether NGcGM3 as an antigen play a role in this process, could be an interesting subject for future studies. We hypothesize, that the simultaneous inhibition of EGFR and uPAR/α5β1 mediated signals, which is induced by the combination treatment on tumor cells, leads to a quite effective form of immunogenic cell death. Dying tumor cells will be a source of NGcGM3 and EGFR together with some strong immune stimulatory signals. The NGcGM3 could be presented on CD1 molecule to directly stimulate NK1.1 cells [46], while peptides derived from EGR will be presented in MHC-I to stimulate CD8^+^T cells [31].

Last, but not least, it shall be noticed that the synergism of the combination provides multiple therapeutic advantage into the human setting. We report a positive clinical outcome for a cancer patient treated with the combination of an anti-EGFR mAb (Nimotuzumab) and anti-NGcGM3 therapy (NGcGM3/VSSP vaccine). Previous studies have shown a relatively high frequency of co-expression of EGFR and NGcGM3 antigen in several human tumors, i.e particularly on lung cancer patients more than 50% of the tumors are double positive [28]. Furthermore, the double expression of these two molecules alone, but even more when combined with EGF expression, correlates with bad prognosis on NSCLC patients [21]. These facts combined to the low toxicity of anti-EGFR mAb, Nimotuzumab [47] and the NGcGM3/VSSP vaccine [22], justify further exploration in clinical trials of the combination. Our preclinical data provide a scenery to explore this combination at least in NSCLC (as our 3LL-D122) and in breast cancer (as our 4T1) tumors. Future studies focused on this combination in other tumor models/localizations with co-expressed targets are needed.

## MATERIALS AND METHODS

### Cell lines

Lewis lung carcinoma (3LL-D122), breast adenocarcinoma (4T1) and murine myeloma P3-X63-Ag8.653 (X63, ATCC, CRL-1580) were cultured in DMEM: F12 (Life Technologies Inc.) supplemented with 10% fetal bovine serum (FBS).

### Lung metastasis models

Female mice 6-to 8 weeks old (18-20g) were purchased from the Center for Laboratory Animal Production (CENPALAB, Havana, Cuba). Animal procedures were performed in accordance with the guidelines stipulated by Animal Committee Review Board of the CIM. Metastasis model 3LL-D122: Spontaneous variant: C57BL/6 mice were inoculated with 2×10^5^ of 3LL-D122-clone into the right hind footpad. Tumors were measured with a caliper. Primary tumors were surgically removed when they reached a diameter of 8-9 mm. Experimental variant: cells were inoculated into the lateral tail vein. 4T1: BALB/c mice were transplanted subcutaneously (s.c.) into the mammary gland with 1×10^4^ of 4T1-clone. Primary tumor diameters were measured every 2-3 days and tumor volume (mm^3^) was determined with the following formula= (minor diameter)^2^ × (major diameter)x π/6. To study overall survival (OS), animals were monitored every day until the primary tumor exceeded 20% of the body mass (4T1-model) and the signs of malignancy appeared. In parallel experiments, the mice were sacrificed on the scheduled days: 3LL-spontaneous (52) or experimental (21); 4T1-spontaneous (40). Lung metastasis were quantified by weighing the lungs. Control groups received the phosphate buffered saline (SSFT).

### Monoclonal antibodies

7A7 mAb for murine EGFR [30]. 14F7 mAb against NGcGM3 ganglioside [32]. α5β1 integrin (Chemicon Int.); uPAR, CD4 and CD8 mAbs (Santa Cruz Biotechnology); pEGFR, pStat3, Src and pSrc (Cell Signaling); FAK and pFAK (Abcam).

### Regarding the treatment

3LL-D122 model: 7A7 mAb (56μg intravenously (i.v.)) was administered at days 3, 5, 7, 27, 29 and 31 plus NGcGM3/VSSP vaccine (200μg (s.c)) at days 7, 21, 32 and 45 or 7A7 mAb plus 14F7 mAb (200μg (i.v.)) at days 3, 5, 7, 9, 11 and 13. 4T1-model: 7A7 mAb at days 3, 5, 7, 11, 13 and 15 plus NGcGM3/VSSP vaccine (100μg) at days 7, 21, 30 and 37.

### Flow cytometric analysis (FACS)

Tumor cells were isolated from remaining metastases after treatments. Cells were incubated with appropriate primary antibodies for EGFR, pStat3, α5β1 integrin, NGcGM3, pEGFR, uPAR, Src, pSrc, FAK and pFAK. Then, tumor cells were washed with SSFT and incubated with FITC conjugated secondary antibodies. All incubations were done for 60 min at 4°C. Control samples were prepared with the second antibody alone. The percentage of positive stained cell was determined by FACSan (Becton-Dickinson). The FlowJo 7.6 program was used to analyze a total of 10^5^ cells acquired on every FACS assay.

### *In vivo* CD4, CD8 and NK1.1-depletion study

mAbs to CD4, CD8 or NK1.1 purified from culture supernatants of YTS191 (anti-CD4), YTS169 (anti-CD8) or PK136 (anti-Natural Killer and/or Natural Killer T cells) (ECACC) rats hybridoma were injected intraperitoneally with 1mg of anti-CD4, CD8 [44] or NK1.1 [29]. This dose has been previously shown to deplete more than 98% of the cell subset.

### Immunohistochemistry staining

Tumor sections were fixed and treated with 3% hydrogen peroxide for 30 min at room temperature. The tissues were incubated with anti-CD31 (PECAM-1, BD Biosciences), anti-mouse Ki67 (Clone MIB-5, Dako), 7A7 or 14F7 mAbs followed by LSAB^®^2 System, Peroxidase, DAB (Dako). The apoptosis was evaluated by ApopTag^®^ Plus Peroxidase In Situ Apoptosis Kit (CHEMICON International). Negative controls were performed by substituting primary mAbs for the SSFT. Each staining included a known positive control: for murine EGFR: 3LL-D122 and X63 cells for NGcGM3. Finally, the sections were counterstained with Mayer’s hematoxylin.

### Immunofluorescence staining (IF)

Cryosections from metastatic lungs were obtained by cryostat (SLEE MEDICAL GMBH Co.) and mounted on plus slides. Afterwards, sections were fixed in 4% paraformaldehyde for 20 min at -20°C. Sections were incubated with appropriate primary mAbs overnight at 4°C. The fluorescent systems used to EGFR, pEGFR, pStat3, NGcGM3, uPAR, α5β1 integrin, Src, pSrc, FAk and pFAK was the same to FACS assay. The sections were counterstained with DAPI. Images were analyzed/captured using the Olympus BX51 LTD microscope. Representative images were photographed at 100X magnification.

### Patients

In April 2005, a 50-year-old male patient with retroperitoneal-peripancreatic hemangiopericytoma was treated at the National Institute of Oncology and Radiobiology (INOR) after receiving approved consent by the Ethical Committee of the Institute. He had a history of radio/chemotherapy with Doxorubicin after surgery with no response defined clinically and by imaging studies. The patient was receiving only palliative care at home. Under compassionate basis, at 18 months after the initial diagnosis, he received the anti-EGFR mAb (Nimotuzumab, Nimo) weekly (i.v.) for six weeks followed by the antibody monthly until 2011. After the first six doses of treatment, the patient received five intramuscular doses of NGcGM3/VSSP vaccine at two week intervals (induction therapy), followed by monthly re-immunizations up to 2011. The pain, skin rash and sequential computed tomography (TAC) from tumors, life´s condition and disease were analyzed during 15 years.

### Statistical analyses

All statistical analysis was carried out using SPSS (version 11.5, SPSS Inc.). For survival analysis the Long-Rank test and Kaplan-Meier curves were used. When data was not normally distributed, nonparametric statistical methods were applied. In all cases, four groups were compared and the Kruskal-Wallis test was used. Data were considered significant when p<0.05.

## SUPPLEMENTARY MATERIALS FIGURES


