# Supplementary material for: Synergistic potentiation of the anti-metastatic effect of anti EGFR mAb by its combination with immunotherapies targeting the ganglioside NGcGM3
Source: Oncotarget. 2018 May 8;9(35):24069–80. doi: 10.18632/oncotarget.25290 (PMC5963610; doi:10.18632/oncotarget.25290)
Supplement: Supplementary file 1 [file oncotarget-09-24069-s001.pdf]

# Synergistic potentiation of the anti-metastatic effect of anti EGFR mAb by its combination with immunotherapies targeting the ganglioside NGcGM3

## SUPPLEMENTARY MATERIALS

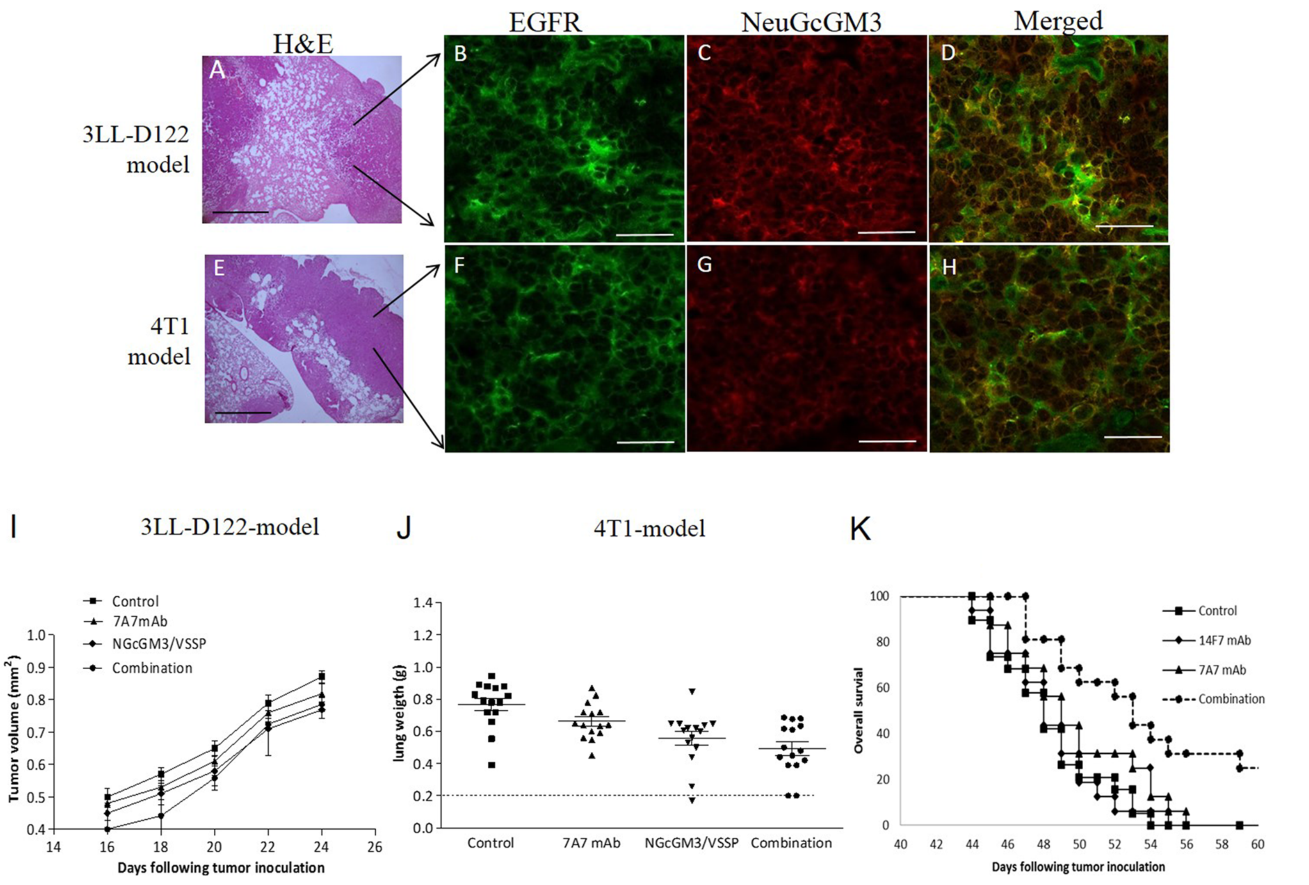


**Supplementary Figure 1: EGFR and NGcGM3 ganglioside are co-localized on spontaneous lung metastasis models.** C57BL/6 mice were inoculated with Lewis Lung carcinoma (2.5×105/mouse) into the right footpad (3LL-D122 model). BALB/c mice were inoculated with the breast carcinoma (1×104/mouse) orthotopically (4T1-model). Histological section of representative lung metastases from 3LL (at day 52, **A**) and 4T1-model (at day 40, **E**) by Hematoxylin and eosin (H&E). Co-localization of both molecules on tumoral section was evaluated by a double immunofluorescence method: 3LL-model **(B-D)** and 4T1-model **(F-H)**. EGFR (green) and NGcGM3 ganglioside (red) positive section. The yellow color identifies co-localization of both molecules on tumor cells (merged) (D and H). Black bars= 20µm, White bars=100µm. The treatment with PBS (i.v.), 7A7 (56µg i.v.), NGcGM3/VSSP vaccine (100 µg (4T1) or 200µg (3LL) s.c.) or Combined therapy is indicated in schematic representation of 3LL (A) or 4T1 models (D) in Figure 1. Primary tumor volume in 3LL-model **(I)**. Each point represents mean±SD of primary tumor/group. Pulmonary metastases were measured as lung weight in 4T1-model **(J)**. Normal lung weight value is indicated (dashes lines). Each group represents mean±SD of lung weight/animals. Passive combinatorial-targeted therapy to EGFR and anti-NGcGM3 therapies increase the survival of mice bearing 3LL-metastasis **(K)**. The animals were treated with PBS (i.v.), 7A7 (56µg i.v.), 14F7 mAb (200µg i.v.) or Combined therapy. To analyze the percentage of survival, animals (n=10/group) of both models were monitored every day. Kaplan-Meier curves of overall are showed (Log-rank test). Statistical analysis was performed using Two-way ANOVA, combined with the Kruskall-Wallis test, for multiples comparison was employed. Statistical differences are indicated: $^{**}p<0.01$, $^{*}p<0.05$. One representative experiment out of three performed experiments is shown in each case.

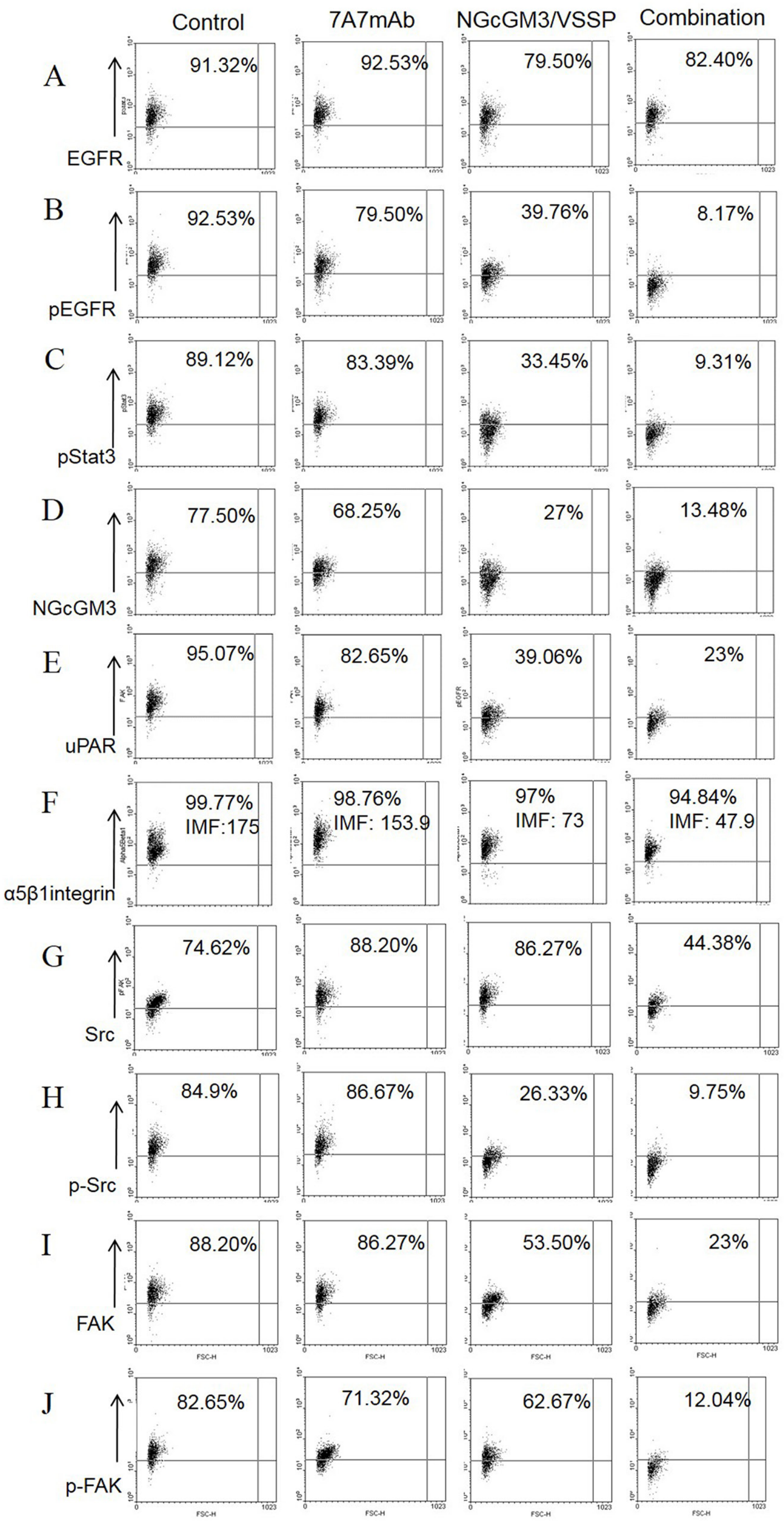


**Supplementary Figure 2: Representative FACS dot plots showing that Combinatorial-targeted therapy to EGFR (7A7 mAb) and anti-NGcGM3 (NGcGM3/VSSP vaccine) simultaneously turns off signaling by the EGFR and uPAR/α5β.** C57BL/6 mice were inoculated with Lewis Lung carcinoma and the treatments as described in Figure 1A. Tumor cells from lung metastasis after treatments were obtained to determine the expression/activation of EGFR **(A)**, p-EGFR (fosfo-EGFR, **B**), p-Stat3 (fosfo-Stat3, **C**), NGcGM3 ganglioside **(D)**, uPAR **(E)**, α5β1 integrin **(F)**, Src **(G)**, p-Src (fosfo-Src, **H**), FAK **(I)** and p-FAK (fosfo-FAK, **J**). Dot plots from one animal is representative of each group. One representative experiment out of three performed experiments is shown in each case.

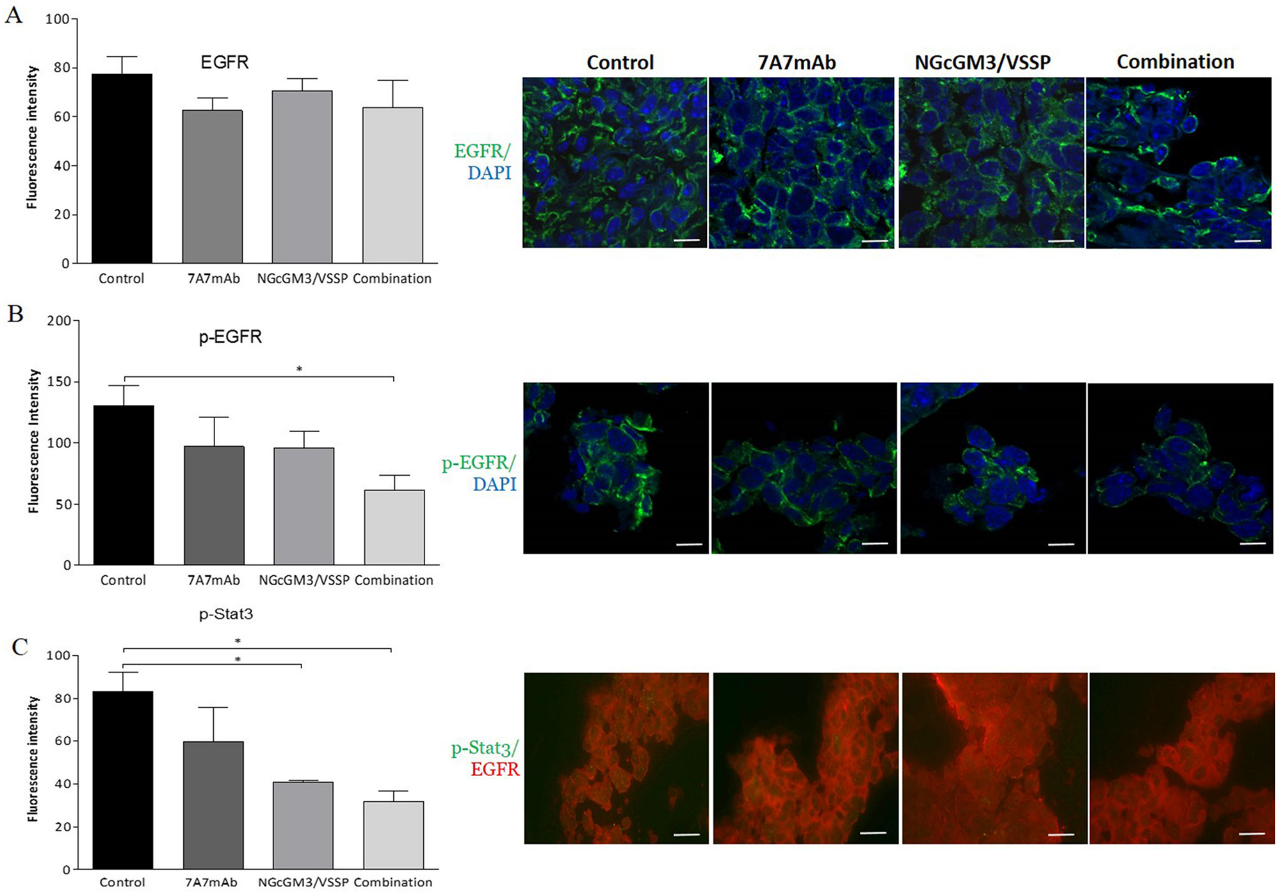


**Supplementary Figure 3: Combinatorial-targeted therapy to EGFR (7A7 mAb) and anti-NGcGM3 (NGcGM3/VSSP vaccine) simultaneously turns off signaling by the EGFR, p-EGFR and pSTAT3 on remaining 3LL-metastases by immunofluorescence.** C57BL/6 mice were inoculated with Lewis Lung carcinoma and the treatments as described in Figure 1A. Tumor sections from lung metastases after treatments were fixed with paraformaldehyde to determine the expression/activation of EGFR **(A)**, p-EGFR (fosfo-EGFR, **B**), p-Stat3 (fosfo-Stat3, **C**). Data are represented as fluorescence intensity and mean±SD (n=6/group). Statistical analysis was performed using Two-way ANOVA, combined with the Kruskall-Wallis test, for multiples comparison was employed. Statistical differences are indicated: $^{*}p<0.05$. One representative experiment out of three performed experiments is shown in each case. White bars=100μm.

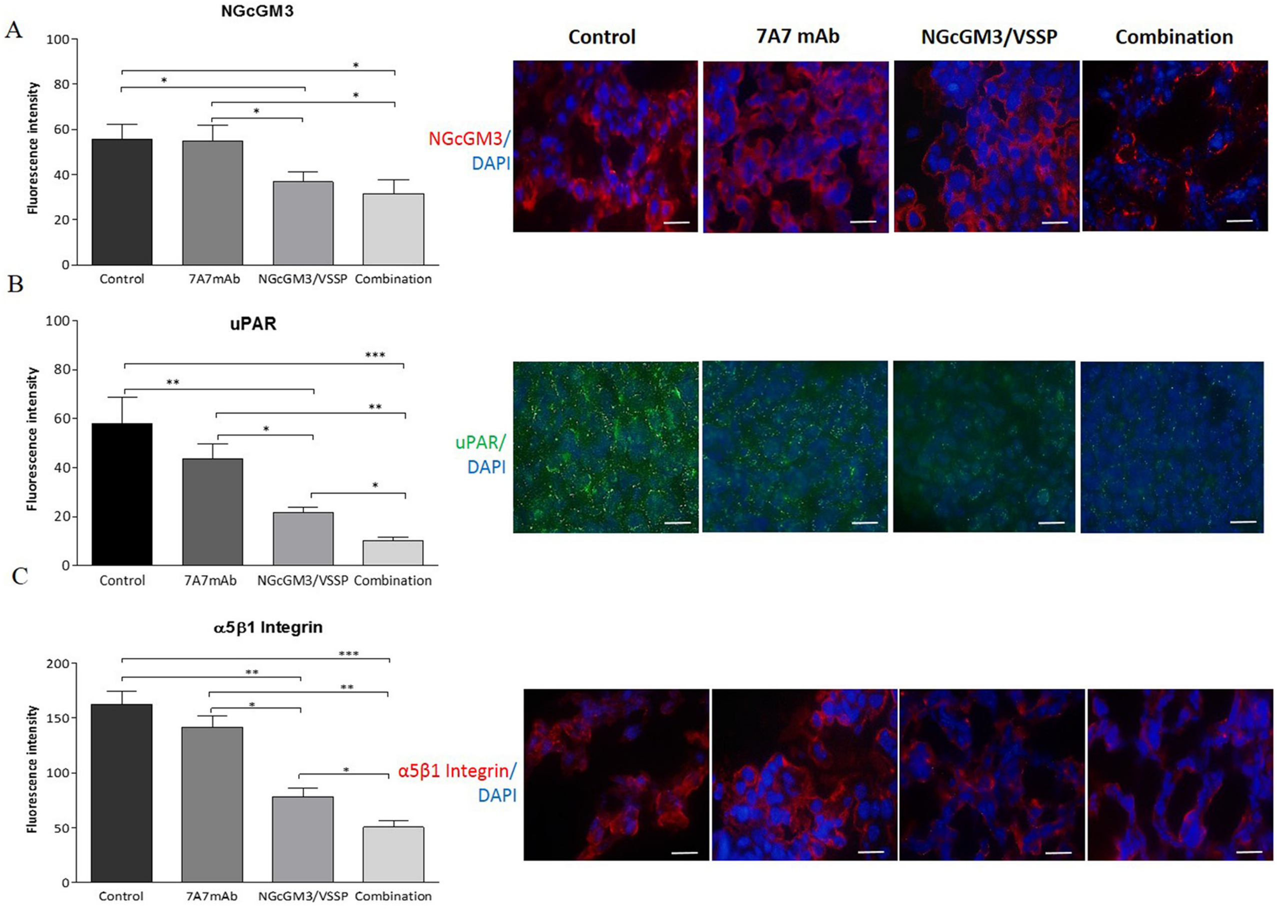


**Supplementary Figure 4: Combinatorial-targeted therapy to EGFR (7A7 mAb) and anti-NGcGM3 (NGcGM3/VSSP vaccine) simultaneously turns off signaling by the uPAR/α5β1 integrin on remaining 3LL-metastases by immunofluorescence.** C57BL/6 mice were inoculated with Lewis Lung carcinoma and the treatments as described in Figure 1A. Tumor sections from lung metastases after treatments were fixed with paraformaldehyde to determine the expression of NGcGM3 **(A)**, uPAR **(B)**, α5β1 Integrin **(C)**. Data are represented as fluorescence intensity and mean±SD (n=6). Statistical analysis was performed using Two-way ANOVA, combined with the Kruskall-Wallis test, for multiples comparison was employed. Statistical differences are indicated: $^{*}p<0.05$. One representative experiment out of three performed experiments is shown in each case. White bars=100μm.

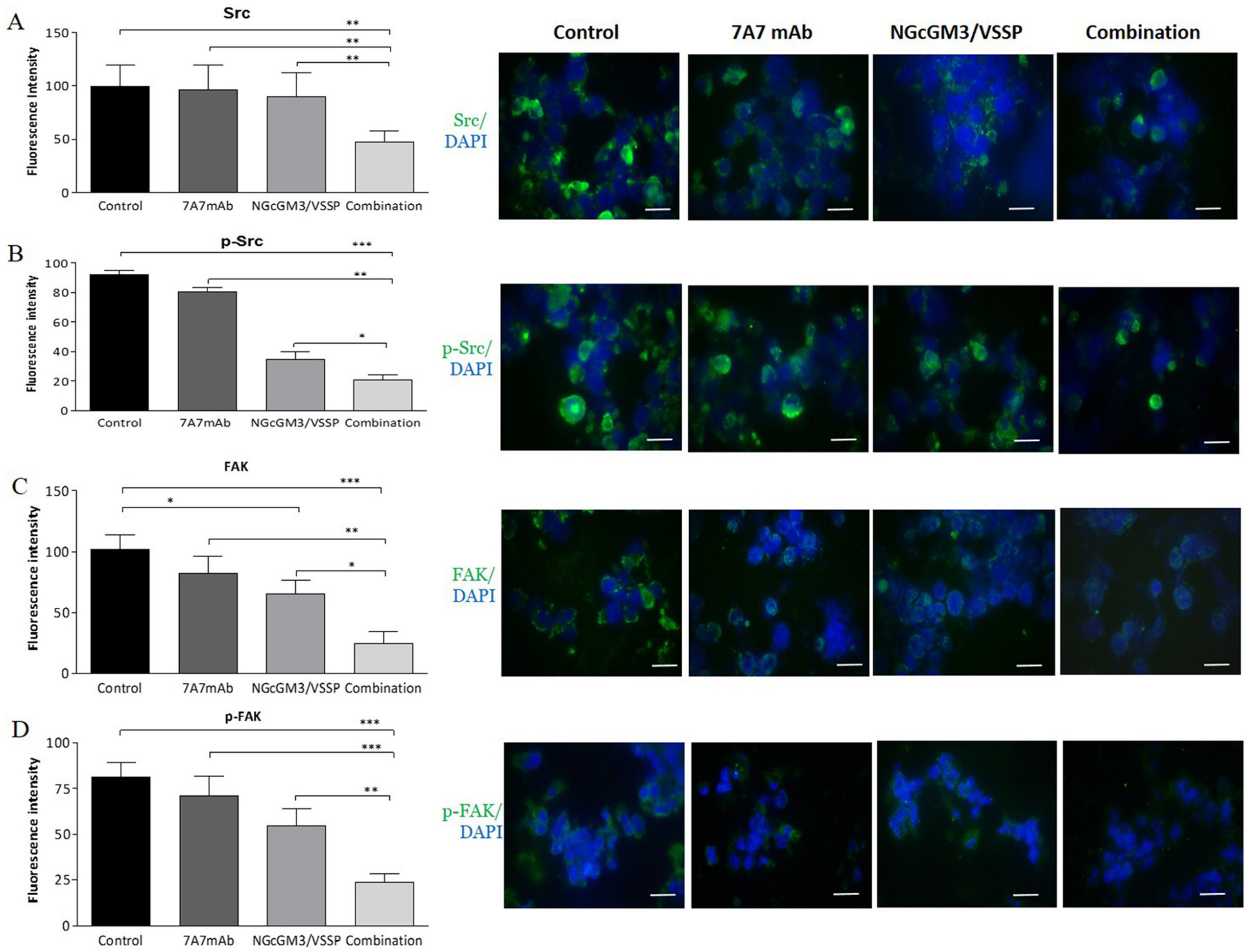


**Supplementary Figure 5: Combinatorial-targeted therapy to EGFR (7A7 mAb) and anti-NGcGM3 (NGcGM3/VSSP vaccine) simultaneously inhibits Src and FAK signaling pathways by immunofluorescence assay.** C57BL/6 mice were inoculated with Lewis Lung carcinoma and the treatments as described in Figure 1A. Tumor sections from lung metastases after treatments were fixed with paraformaldehyde to determine the expression/activation of Src **(A)**, p-Src (fosfo-Src, **B**), FAK **(C)** and p-FAK (fosfo-FAK, **D**). Data are represented as fluorescence intensity and mean±SD. (n=6/group). Statistical analysis was performed using Two-way ANOVA, combined with the Kruskall-Wallis test, for multiples comparison was employed. Statistical differences are indicated: $^{***}p<0.001$, $^{**}p<0.01$, $^{*}p<0.05$. One representative experiment out of three performed experiments is shown in each case. White bars=100μm.

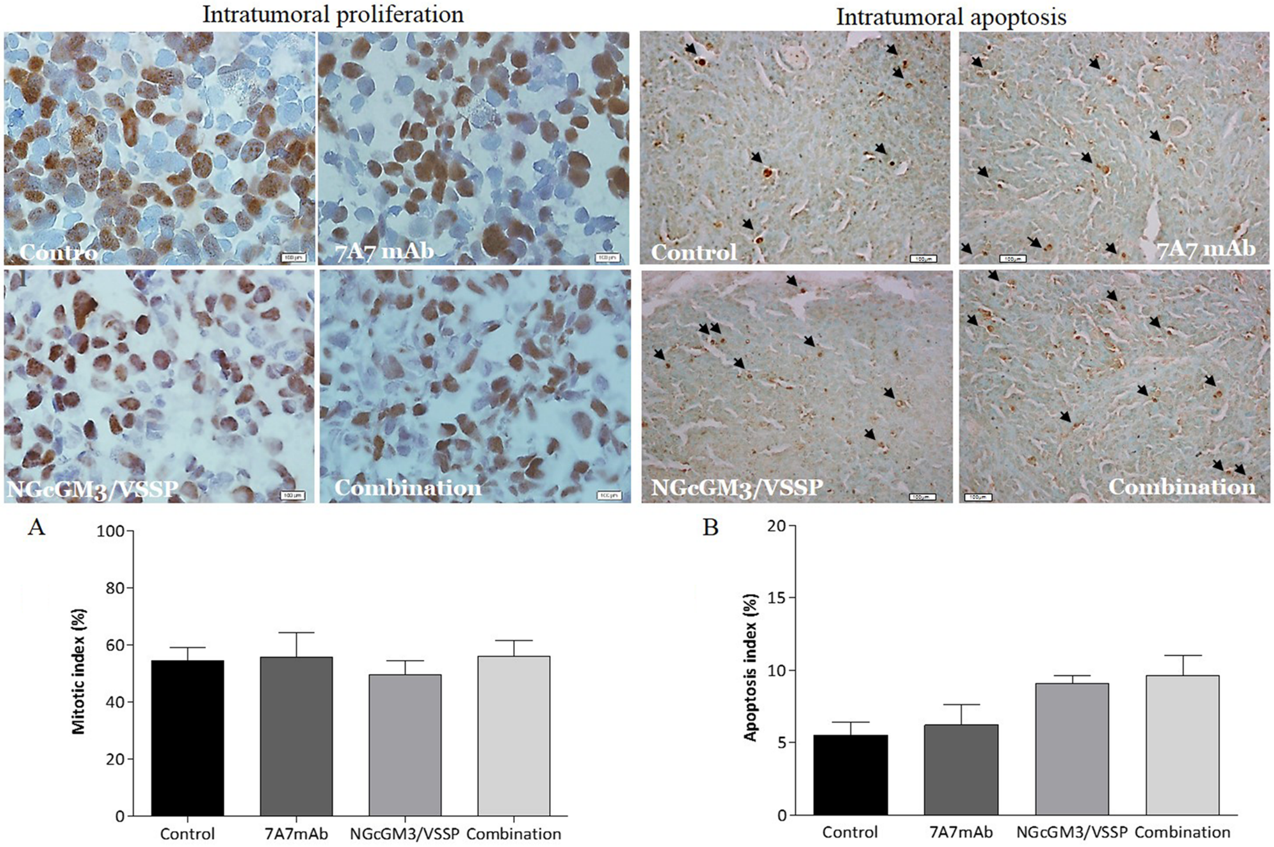


**Supplementary Figure 6: *In vivo*, effects of Combinatorial-targeted therapy to EGFR (7A7 mAb) and anti-NGcGM3 (NGcGM3/VSSP vaccine) on intratumoral proliferation and apoptosis on remaining 3LL-metastases.** C57BL/6 mice were inoculated with Lewis Lung carcinoma and the treatments as described in Figure 1A. Representative image and quantitative results of immunohistochemical staining of cell proliferating cells by Ki67 **(A)** and apoptotic cells by ApoptTag systems method **(B)**. Data represent the mean±SD in ten independent lung metastases fields from five mice/group. One representative experiment out of three performed experiments is shown. Statistical analysis was performed using Two-way ANOVA, combined with the Kruskall-Wallis test, for multiples comparison was employed. Statistical differences are indicated: $^{*}p<0.05$.

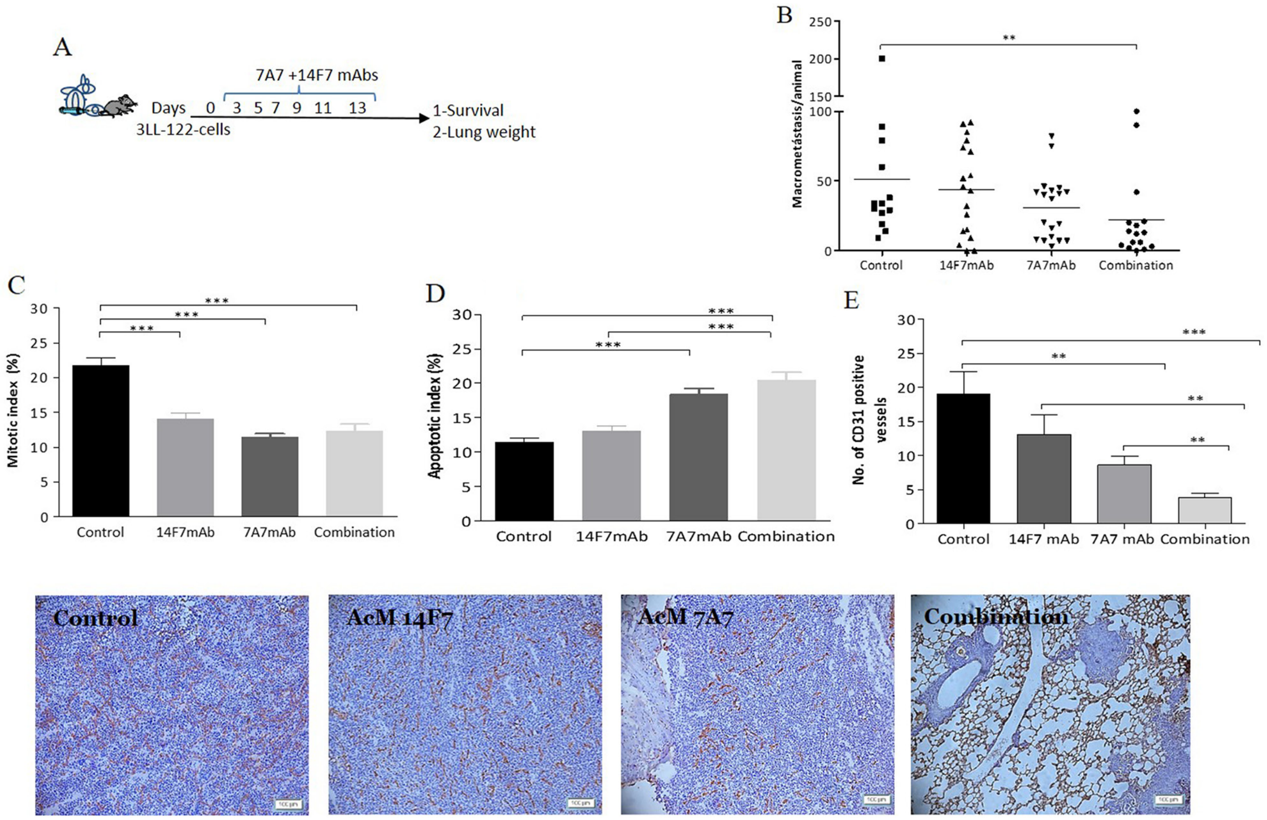


**Supplementary Figure 7:** ***In vivo*** **effect of Combinatorial-targeted therapy to EGFR and anti-NGcGM3 on 3LL tumor-bearing mice.** C57BL/6 mice were inoculated with Lewis Lung carcinoma (2.5×105, i.v.) **(A-E)**. Administration of treatment with PBS (i.v.), 7A7 (56μg i.v.), 14F7 mAb (200μg i.v.) or Combined therapy is indicated in schematic representation (A). At day 21, mice were sacrificed and lung macrometastases were counted (B). Tumor sections from lung were fixed with ice-cold acetone to determine the mitotic index by H&E staining (C). Apoptotic index by the ApoptTag systems method (D). The number of $CD31^{+}$ microvessels by immunohistochemistry assay (E). Representative image of immunostaining of angiogenic activity. Data represent the mean±SD in 10 independent lung metastases fields from five mice/group. Statistical analysis was performed using Two-way ANOVA, combined with the Kruskall-Wallis test, for multiples comparison was employed. Statistical differences are indicated: $^{***}$p<0.001, $^{**}$p<0.01. One representative experiment out of three performed experiments is shown in each case.

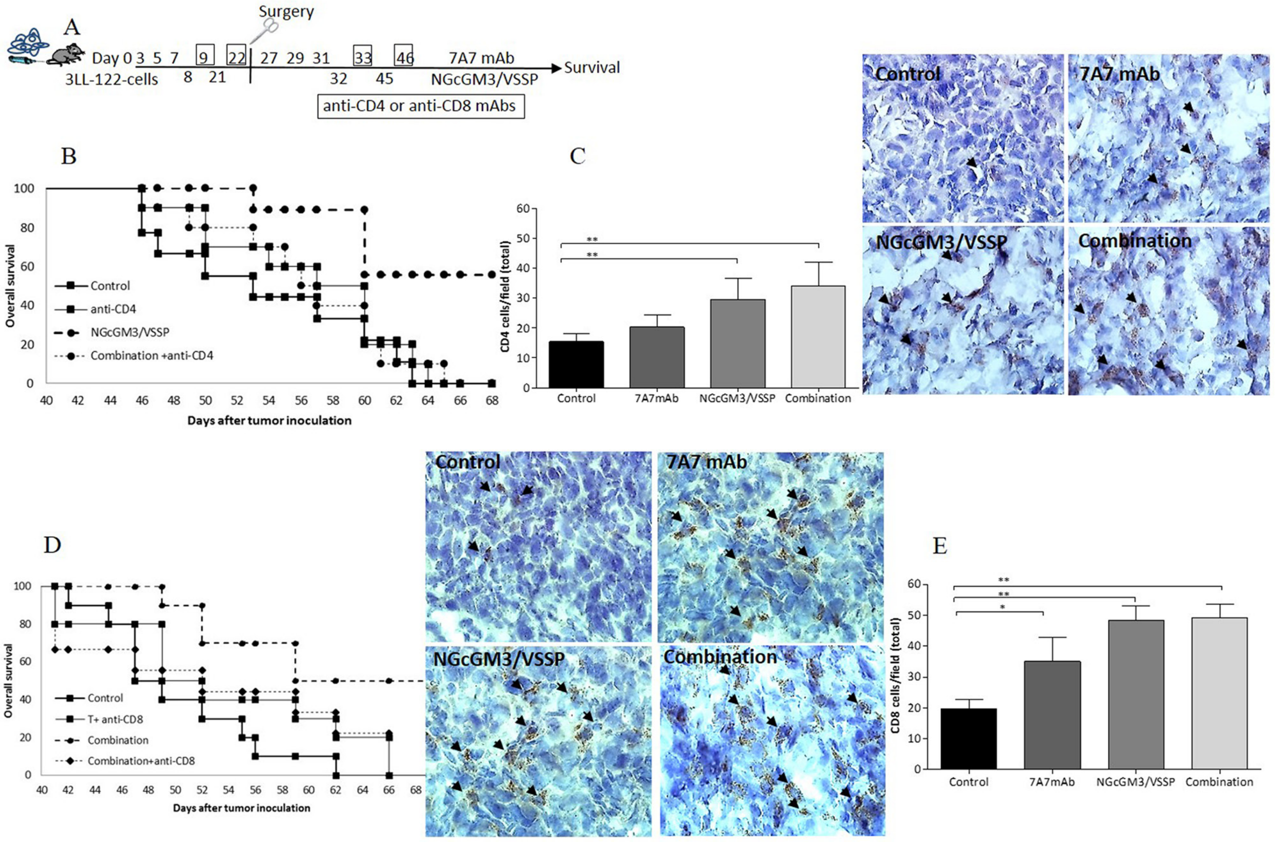


**Supplementary Figure 8: Involvement of cellular immune in the increased survival by the combination therapy.** C57BL/6 mice were inoculated with Lewis Lung carcinoma (2.5×105/mouse) into the right footpad (A). Administration of treatment with PBS (i.v.), 7A7 (56µg i.v.), NGcGM3/VSSP vaccine (200µg s.c.), Combined therapy or CD4$^+$T and CD8$^+$T cells depletion is indicated in schematic representation **(A)**. To analyze the percentage of survival, animals (n=10/group) were monitored every day. Kaplan-Meier survival curves are showed for CD4$^+$T cells (**B**, p<0.028) or CD8+T cells (**D**, p<0.032) depletion. Representative photomicrographs of lung 3LL-metastases showing the CD4$^+$T and CD8$^+$T cells infiltration by the immunohistochemistry staining. Quantitative evaluation of CD4$^+$T cells **(C)** and CD8$^+$T cells **(E)**. Statistical analysis was performed using Two-way ANOVA, combined with the Kruskall-Wallis test, for multiples comparison was employed. Statistical differences are indicated: $^{**}$p<0.01, $^{*}$p<0.05. One representative experiment out of three performed experiments is shown in each case.
